# The UK COVID‐19 app: The failed co‐production of a digital public service

**DOI:** 10.1111/faam.12307

**Published:** 2021-10-11

**Authors:** Tobias Polzer, Galina Goncharenko

**Affiliations:** ^1^ Institute for Organization Studies WU Vienna University of Economics and Business Vienna Austria; ^2^ Department of Accounting and Finance University of Sussex Business School, University of Sussex Brighton UK; ^3^ Department of Accounting, Auditing and Law NHH – Norwegian School of Economics Bergen Norway

**Keywords:** accountability, contact tracing apps, co‐production, digital technologies, digital transformation, netnography

## Abstract

The COVID‐19 pandemic put governments under pressure to make radical and urgent decisions, and to implement new digital solutions to steer society and deliver public services. Our study analyzes social media discourse to understand the co‐production of a digital public service in an emergency situation. Empirically, we mobilize Twitter netnography and discourse analysis to examine citizens’ perceptions of the contact tracing app (CTA) introduced by the UK government to tackle the pandemic and save lives. Our study contributes to research on public sector accountability for digital transformations by advancing scholarly understanding of how societal concerns and public perceptions impact the co‐production of digital services. Our findings reveal a high level of public skepticism toward the app and a general distrust of the UK government among the main social challenges of the CTA's implementation. Furthermore, we evidence widespread public distress over the potential violation of democratic freedoms and misuse of the data collected by the app. Finally, we reflect on the linkages between the lack of governmental accountability and the difficulties in mitigating the expressed societal concerns, causing a corresponding resistance on the part of the public to engage in and support co‐production.

## 1 INTRODUCTION

The introduction of digital technologies to the delivery of public services reshapes the interactions between governments and citizens (Agostino et al., 2020). Public sector organizations are transforming their operations and activities, adapting to the increasing presence and pressures of digitalization (Mergel et al., 2019). Today's governments actively engage with digital solutions for service delivery, accountability and co‐production with various stakeholders. At the same time, progress in co‐production and the corresponding intensification of stakeholder dialogue, that is, the “reciprocal engagement and collective cognition” (Payne & Calton, 2002, p. 38), enhance public scrutiny of government actions and social debates.

The extraordinary circumstances of the COVID‐19 pandemic have offered new settings to explore the unique intersection between the provision of digital services and co‐production in the midst of an emergency. The pandemic brought governments into situations beyond “business as usual” and forced them to mobilize instruments and resources to make radical decisions under extreme pressures of urgency and public scrutiny. The governments that were able to combine their management skills with acceptance of measures by citizens were the most successful in their responses to the pandemic (Christensen & Lægreid, 2020). Co‐production played a vital role, as many public government policies only worked because citizens co‐operated voluntarily and on a large scale, for example by co‐producing medical goods and services and adhering to government lockdown policies (Steen & Brandsen, 2020).

However, so far scholarly understanding of the co‐production of public services during emergencies is limited (Steen & Brandsen, 2020). In particular, little is known about the potential side effects of collaborative governance mechanisms during a pandemic (Jayasinghe et al., 2020), and how the momentum of voluntary co‐production can be kept up (Steen & Brandsen, 2020). Prior research on digital co‐production offers the Unified Theory of Acceptance and Use of Technology (UTAUT) mainly used to study the acceptance of digital public services in terms of the technology itself (e.g., Saxena & Janssen, 2017; Wirtz et al., 2019). However, citizens’ acceptance and willingness to engage, eventually driving the effectiveness of digital technologies, goes beyond these formal‐rational aspects (Hirschheim, 2007). Therefore, critics of UTAUT hold that the salient perceptions of users and the role of the social environment in technology acceptance are not adequately recognized by this theory (Benbasat & Barki, 2007). This gap extends to the wider implications of digital technologies in forging collaboration and engagement with citizens (Agostino et al., 2021).

Considering the complexities of the social environment that impact technology acceptance as well as the rational aspects discussed by UTAUT, our study addresses the research question of how citizens’ perceptions affect the co‐production of digital technologies in the public sector during an emergency. We carry out a discourse analysis of the public discussion of the contact tracing app (CTA) introduced in the UK during the COVID‐19 pandemic. We understand discourse as a “form of language‐based verbal [and written] communication produced as a means to generate a response from the […] population” (Dubnick, 2014, p. 29). Discourses are insightful indicators of public perceptions as they create commonly accepted narratives, “provide individuals with subject positions” (Cassell & Symon, 2004, p. 205) and capture a comprehensive picture of social reality (Dick & Cassell, 2002).

Our study uses the methods of netnography (Kozinets, 2015) and discourse analysis (Atkinson, 2017; Cassell & Symon, 2004; Dick & Cassell, 2002) to capture the contextual richness of the public discussion about the UK CTA on Twitter. As the country in Europe most severely affected by the COVID‐19 pandemic at the time of our study, the UK provides a fertile empirical site to observe digital service co‐production as a key governmental response to an emergency. In particular, the phased introduction of the UK's CTA and the corresponding complexities kept it in the spotlight of public attention, amplifying the polarization of opinions.

Our study contributes to research on accountability for digitalization of public services (Hood & Dixon, 2016; Lapsley & Miller, 2019) in multiple ways. First, we deploy UTAUT in the contemporary setting of a public sector emergency and evidence the importance of social perceptions in foreshadowing the outcome of the digital service co‐production. Second, our study refines our understanding of the role of accountability in public sector co‐production by revealing how poorly addressed demands for governmental accountability can lead to the failure of co‐production despite the initial willingness of citizens to engage. Finally, we advocate that scholarly research moves beyond studying the co‐production of each digital public service in isolation and broadens its scope by acknowledging the impact of social complexity, wider political developments, and strategic public policies, debates and discourses (Cooper & Lapsley, 2019).

The remainder of the study is organized as follows. The next section presents the theoretical grounds of the study. Section 3 outlines the empirical context of the CTA introduction in the UK during the COVID‐19 pandemic. Section 4 explains the chosen research method and the research design. Section 5 presents the main findings of the study. We finish by discussing the implications of our findings and providing concluding remarks in Section 6.

## 2 THEORETICAL BACKGROUND

To explore the theoretical background to our study, this section engages with two streams of knowledge: first, current understanding of co‐production between public sector organizations and citizens (2.1). Second, the social aspects of co‐production, paying attention to the notions of trust, accountability and social environment (2.2).

### 2.1 Co‐production of public services

As a manifestation of collaborative governance and democratic participation, co‐production in the public sector has received substantial scholarly attention (Bovaird & Löffler et al., 2021; Cepiku et al., 2020; Dudau et al., 2019; Nabatchi et al., 2017; Osborne et al., 2021; Voorberg et al., 2014; Zhao & Wu, 2020). Co‐production has been defined as the “voluntary or involuntary involvement of public service users in […] design, management, delivery and/or evaluation of public services” (Osborne et al., 2016, p. 640). Areas where co‐production is nowadays frequently used include customer journey mapping in service design, deliberative participation in urban planning and volunteering in elderly care. Some definitions stress its normative aspect in the sense that it is hoped that co‐production would lead to “better value,” for example with respect to a higher quantity or quality of services (Dudau et al., 2019). Building on this “value perspective,” Osborne et al. (2021) argue that research needs to consider both sides of the production, as well as the consumption and use of services. The distinction between voluntary and involuntary (or coercive) co‐production (Tõnurist & Surva, 2016), however, prompts questions of power relationships between governments and involved service co‐producers, acknowledging the dominant position of governments in the process (Steen & Brandsen, 2020; Turk et al., 2021).

In the situation of continuing public sector austerity, co‐production has been seen as a way to access the resources of society (Brandsen & Honingh, 2016), for example when public agencies transfer the responsibility of care onto families, neighbors and friends. Co‐production's supporters argue that it allows the development of targeted solutions around users’ needs, leading to increased user satisfaction, efficiency gains and lower government personnel costs for delivering services (Tõnurist & Surva, 2016). In addition, its proponents suggest, the “local ownership” of co‐produced services enhances the capacities and, eventually, the confidence of individuals and wider communities, such as in participatory budgeting. Ultimately, co‐production is thought to strengthen cohesion in a fragmented society by democratizing the public sector (Brandsen & Honingh, 2016) and amplifying citizens’ trust in governments (Fledderus et al., 2014).

In an era of digital government, co‐production extends to the use of crowdsourcing, open data and social media (Lember et al., 2019; Meijer, 2012). It has been found that “new media not only shift co‐production away from a rational approach to a more social approach, but also strengthen the emphasis on social […] interactions” (Sorrentino et al., 2018, p. 283). But research has also referred to a “dark side” of co‐production, that is, dysfunctional aspects, unintended consequences and even the co‐destruction of value (Dudau et al., 2019; Jayasinghe et al., 2020; Loeffler & Bovaird, 2019). Projects can fail when co‐production is done ineffectively (e.g., due to the incongruence of parties’ values or a lack of skills), illegal processes are involved (e.g., discrimination or infringement of privacy) or public governance principles (e.g., accountability, transparency, equality or due process) are neglected (Loeffler & Bovaird, 2019). To better understand the wider governance principles of co‐production (e.g., Dudau et al., 2019), we now turn to the social aspects of co‐production.

### 2.2 The UTAUT and the social aspects of public service co‐production

Services, regardless of whether they are offered in the private or public sector, require the involvement of potential users, their acceptance of technology and active engagement (Osborne et al., 2016). If citizens will eventually co‐produce depends on the context‐specific conditions that shape the sensibility of ideas, the plausibility of solutions and the feasibility of claims made by governments (Alford, 2002).

A number of factors contribute to digital services being accepted and (eventually) used. Previous research on the acceptance of new technologies has formulated a UTAUT, which identified four factors as significant for the acceptance: *performance expectancy* refers to the degree to which a person believes that an innovation will be helpful to getting a certain task done; *effort expectancy* describes how easy or difficult it is for an individual to use a technological innovation; the *facilitating conditions* are concerned with whether an individual is thinking that a supporting technical infrastructure is in place if difficulties occur; *social influence* refers to the ease with which somebody's peers find it to utilize a new service or product (Venkatesh et al., 2003). While UTAUT was not designed for a particular sector, this theory has previously been used to research the acceptance of digital services in the public sector (Saxena & Janssen, 2017; Wirtz et al., 2019).

Despite its acclaimed utility in examining users’ perceptions of digital services, UTAUT has been challenged on a number of grounds (Hirschheim, 2007). For instance, the theory has been criticized for being overly simplistic and rational‐mechanistic, failing to capture important aspects of the social environment. Other criticisms relate to the lack of consideration of salient beliefs of users (Benbasat & Barki, 2007) and their wider concerns about technological solutions as potential instruments of control (Kellogg et al., 2020). Finally, it is argued that UTAUT does not adequately recognize aspects of the notion of trust (Gefen et al., 2003). Therefore, to supplement the use of UTAUT in understanding the co‐production of digital public services within the complex setting of contemporary social developments, it is essential to acknowledge and explore the role of wider social aspects.

We approach this by highlighting that the notions of trust, accountability and social environment are key to understanding the settings in which co‐production takes place. At the same time, we recognize that these concepts aim to encapsulate dynamic and multifaceted phenomena (Bovens, 2007; Dubnick, 2014; Goncharenko, 2020; Mulgan, 2000; ter Bogt & Tillema, 2016). Our intention here is to explore how the concepts of trust, accountability and social environment could complement UTAUT in for revealing contextual richness and in developing an understanding of what it really means when citizens are invited to co‐produce with their governments. This also explains our scholarly interest in (online) public discourses as “interpretive repertoires […] being used to construct certain accounts of reality […] [and] produce different versions of social practice” (Cassell & Symon, 2004, p. 206). Public discussions, therefore, play a crucial role in the success or failure of government initiatives, by mobilizing consent or disagreement.

(Public) trust has been defined, in essence, as “a belief in the reliability, truth or ability of something or someone” (Hyndman et al., 2021, p. 3). Research has acknowledged the dual perspective of trust in the government, which can be considered as both an outcome for co‐production and a prerequisite. On the one hand, co‐production is said to serve as a vehicle to restore and increase trust in government (Brandsen & Honingh, 2016). On the other hand, pre‐existing trust of citizens in the government is a necessary condition in the first place to enable co‐production (Fledderus et al., 2014). Trust encourages collaboration and allows individuals to rely on one another's actions (Yates et al., 2021). In his conceptualization of trust, Luhmann (2018) emphasized its relational nature, as trust in somebody or something cannot be conferred without prior experience of that somebody or something. Therefore, the level of trust that people have in a government is influenced by its previous actions. This makes the social environment, such as accumulated conditions and settings of past collective experiences of interacting with the government, a relevant factor in co‐production (Coletti et al., 2005; Mansoor, 2021; Van De Walle & Six, 2013).

The concepts of trust(worthiness) and accountability are linked to each other as both “have the potential to underpin each other to create a “virtuous circle” (Hyndman et al., 2021, p. 7). Like trust, accountability, that is the process to hold someone to account for their actions (Mulgan, 2000), is a particular aspect of the social environment (ter Bogt & Tillema, 2016; Van De Walle & Six, 2013). Therefore, (digital) public services that governments propose and deliver directly to citizens are expected to demonstrate improvements in public accountability. This direct engagement in the form of service delivery amplifies the importance of accountability conduct for successful digital transformation in the public sector (Pina et al., 2007). Trust defines a system of accountability between involved parties (Yates et al., 2021); regularly exercised (mutual) accountability enhances the level of trust (Goncharenko, 2020). In the hectic environment of an emergency such as the COVID‐19 pandemic, governments seek to (re)gain public trust through structuring their accountability conduct. This can manifest in framing stakeholder dialogue and public discourse around emergency solutions that have been proposed to establish a new normalcy and to reinforce social consciousness (Cooper & Lapsley, 2019; Ferri & Zan, 2019).

To conclude, the theoretical concepts we mobilize in our study acknowledge the importance of both the technical and the social aspects of the co‐production of digital public services. While the well‐established UTAUT stresses rational‐technical factors, there is also a need to contextualize the application of this theory in digital co‐production, as the social environment defines the initial strands of co‐production and accountability relationships between the parties. For emergency digital technologies to succeed, it is therefore paramount to understand how social aspects and citizens’ perceptions manifest in public discussions and impact co‐production. The next section presents the empirical context in which we study this important question.

## 3 EMPIRICAL CONTEXT

At the beginning of 2020, governments across the globe proposed lockdowns in the name of public interest and the protection of public health and safety during the COVID‐19 pandemic. Once infection rates decreased, often a phased approach was taken to re‐open economies, facilitated by “route maps” and systems of “alert levels” (Cellan‐Jones, 2020). A substantial effort was put into such planning exercises, as governments dealing with the pandemic faced a number of challenges around the re‐opening of economies.

Technology facilitated governments’ strategic planning of their responses to the pandemic. The pandemic also accelerated developments in the digital transformation of government services (Agostino et al., 2020). To fulfil the objective of re‐opening economies and return to “new” social normalcy while controlling COVID‐19 transmission, CTAs were introduced as an integral technological component of governmental containment strategies (Ferretti et al., 2020). CTAs are constructed to “track whom each user has been in proximity to and can then alert all affected users when one of them confirms positive for infection” (Farronato et al., 2020).

CTAs serve as an example of both voluntary and involuntary forms of co‐production, depending on the country. While the use of CTAs is voluntary in most Western countries, they have been intensively promoted by a number of provincial governments in China (Cheng et al., 2020) and their use has even been made mandatory in Qatar (Jacob & Lawarée, 2020). If digital contact tracing is to be delivered effectively, a substantial number of citizens have to download, install and run the app—that is to co‐produce the service (Farronato et al., 2020). CTAs can only achieve a positive outcome if adoption rates are high, at around 60% of the population at least (Findlay et al., 2020).

The UK government embarked on a project to develop its CTA in the midst of the first pandemic lockdown in March 2020. The CTA became central to the government's strategy to deal with the pandemic and, eventually, to ease lockdown restrictions (Cellan‐Jones, 2020). Two models have been developed. Under the so‐called “centralized approach,” the anonymized gathered data is transmitted to a remote server where matches are made with other contacts when a user develops symptoms of the illness. By contrast, in the decentralized approach, users are given more control over their information (by keeping it on the phone) and the matchmaking is done on the phones as well (Kelion, 2020).

A framework that takes the latter approach was jointly developed by technology companies Apple and Google (Kelion, 2020). As well as offering enhanced privacy, this approach has been claimed to work better with certain phones than the centralized system (ibid.). NHSX, the innovation unit of the National Health Service (NHS), was in charge of delivering the UK's CTA. An initial version—taking the “centralized” approach—was developed and trialed on the Isle of Wight in May 2020 (Kelion, 2020). Parallel to the trial, a debate emerged as to whether a “decentralized” or a “centralized” approach to contact matching would be more appropriate. After the trial, a shift to the “decentralized” approach was made, using the Apple/Google framework (Kelion, 2020), causing a delay to the introduction of the final version.

Various potential strategies for governments in the UK and beyond to increase the acceptance of CTAs have been proposed (Farronato et al., 2020). First, instead of trying to penetrate the whole society, roll‐out could begin in contained communities where CTAs would be instantly useful. Second, CTAs need to demonstrate a value added for users—such as a function in the software for checking potential symptoms of COVID‐19 and information on the situation in the local area. Third, adoption rates can be improved by offering CTA users additional services—such as cheaper or free testing (Farronato et al., 2020). Such tactics resonate with findings from e‐government research that identifies two antecedents significant for citizens’ intentions to use mobile government services: perceived ease of use and usefulness of services (Wirtz et al., 2019).

Moving beyond the functional level, due to the necessarily invasive character of CTAs (Privacy International, 2020), other aspects are equally important. The extent to which citizens perceive mobile digital government services as secure and protective of their privacy is crucial for the willingness to use e‐government services (Wirtz et al., 2019). Looking at the Health Code app that monitors the health status related to COVID‐19 of every citizen in one of the Chinese provinces, Yang (2020, p. 662) concludes that such apps, “as well as other solutions based on big data technologies, raised concerns over mass surveillance, privacy and information security.” Therefore, governments are challenged by the need to balance public interest and access to data with privacy (Wirtz et al., 2019). In short, CTAs cannot succeed without the populace's general trust in the government (Cairney & Wellstead, 2020).

Our study aims to capture these empirical developments from the perspective of citizens, and in particular their perceptions of the proposed co‐production, as manifested in the individual reactions revealed on social media. First, we will outline our methodology and reasons for choosing it.

## 4 METHODOLOGY

The lockdowns imposed by governments amplified the salience of digital and social media platforms for citizens to speak out and communicating views on governments’ emerging responses to the pandemic. Acknowledging this role of social media in facilitating discourse (Bellucci & Manetti, 2017; Mansoor, 2021), our study mobilizes the methods of netnography (Jeacle, 2020; Kozinets, 2015) and discourse analysis (Cassell & Symon, 2004; Dick & Cassell, 2002; Duval & Gendron, 2019) to examine public perceptions of the CTA introduction. This section discusses the research approach and design of our study.

Social media enables human interaction (Bellucci et al., 2019; Gallhofer et al., 2006). Speaking arenas can be regarded as self‐established accountability forums responding quickly to shifts in economic and political agendas and societal imperfections (Neu et al., 2019) and having the potential to overcome the administrative barriers associated with traditional accountability channels (Jeacle & Carter, 2011, 2014). Twitter is one of the most popular contemporary speaking platforms on which to express opinions and contribute to discussion (Neu et al., 2019). Even though other online platforms, such as Facebook and LinkedIn, also host an eclectic assemblage of viewpoints, the Twitter arena is characterized by its transparency (all Twitter accounts and tweets are public) and concise statements (a Twitter post cannot exceed 280 characters) (Neu et al., 2019).

Accounting research acknowledges the informative richness of social media data, its ability to construct and communicate “accounts” and “counter accounts” and reflect the overall public sentiment (Duval & Gendron, 2020; Gallhofer et al., 2006). Equipped with a corresponding research method called netnography (Bellucci & Manetti, 2017; Jeacle, 2020), scholars have examined the role of social media discourses in the areas of dialogic accounting, stakeholder engagement, social reporting and accountability (Bellucci et al., 2019; Jeacle & Carter, 2011, 2014; Neu et al., 2019). Netnography allows the researcher to observe textual discourse, while preserving the authentic nature of discussions created and shared freely by numerous contributors (Bellucci & Manetti, 2017; Jeacle & Carter, 2011). The method assists in the understanding of social phenomena by the identification of themes, patterns and propositions. Netnography is grounded in the principles of theoretical sampling and theoretical generalization, in contrast to systematic sampling and statistical generalization (Eisenhardt, 1989; Kozinets, 2015).

We mobilized netnography to build a comprehensive dataset to analyze public discourse on the UK CTA introduction. As Twitter continuously produces massive amounts of data containing the opinions freely expressed online, we developed a multifaceted approach to navigate within the flow of information. Table 1 outlines the three stages of data collection, coding and analysis.

**TABLE 1:** Research design: Data collection, coding and analysis

Stage 1: Government‐to‐citizens	
Objective	‐Exploration of citizens’ reactions to government organizations announcing the introduction of the UK CTA on Twitter ‐Identification of themes and patterns of citizens’ reactions
Approach	Collect tweets, retweets and comments of citizens from official Twitter accounts of government organizations introducing CTAs
Data acquired	Five Twitter discussions initiated by: ‐@10DowningStreet (“United Kingdom government account. Official page for Prime Minister @BorisJohnson's office, based at 10 Downing Street”) ‐@NHSX (“leading the digital transformation of health and care”) – Two threads ‐@NHSTestTraceapp (“official account for […] #NHSTestandTraceapp”) ‐@iwight (“official updates from the Isle of Wight (UK) Council”)^1^ containing 1188 retweets and comments within 342 subdiscussions
Stage 1 analysis	‐Coding of contents of acquired data (unit of analysis: individual tweet): 384 assigned codes ‐Systematization of the assigned codes into 15 initial themes: privacy concerns, technical issues, (dis)trust (skepticism), (de)centralization, surveillance, effectiveness, efficiency (usability), installation, transparency, security, corruption, procurement, control, (democratic) freedoms and misuse
Stage 2: Citizens‐to‐citizens	
Objective	‐Acknowledgment of the voices of citizens ‐Exploration of the data richness and theme identification beyond Stage 1
Approach	Performing 30 searches with the keywords “corona app” or “tracing app” accompanied by keywords from one of the 15 initial themes identified during Stage 1 and the country (UK) – for instance, “corona app transparency UK,” “tracing app security UK”.
Data acquired	1014 tweets within 205 Twitter discussions
Stage 2 analysis	‐Coding of acquired data (unit of analysis: individual tweet): 396 assigned codes ‐Confirmation of the initial themes from Stage 1 and achievement of empirical saturation ‐Identification of five additional initial themes: individual choice, coercion, interoperability, delay (unfulfilled commitment), mission drift
Stage 3: Analysis, interpretation and theorization	
Objective	‐Creation of an integrated understanding of topics ‐Contextualization of themes and evoking the “big picture”
Approach	‐Multiple rounds of analysis ‐Identification of aggregated topics
Data	Stages 1 and 2 datasets combined
Outcome	Systematization of the theme codes into eight aggregated topics: “performance and security,” “effectiveness,” “(de)centralization,” “individual choice and usability,” “privacy and surveillance,” “accountability and transparency,” “(dis)trust” and “freedom and misuse” (see Table 2)

*Data*: Considering Twitter as a public discussion arena, we identified two types of discourses relevant to our study. The first one, “government‐to‐citizens” is related to the initial introduction of the UK CTA by four governmental organizations using their official Twitter accounts. Here, we were interested in the Twitter responses of citizens to such announcements within the same Twitter threads. For instance, when the trial version of the app was released, the following message was published on the account of the UK Prime Minister (triggering 206 reactions):

“If you live on the Isle of Wight I have a simple message: Download the app to protect the NHS and save lives.” – @MattHancock [Secretary of State for Health and Social Care] announces the launch of the NHS track and trace app on the Isle of Wight, ahead of its roll‐out across England (@10DowningStreet; Tweet ID #1257595048415449088).

We performed the Stage 1 data collection and initial analysis around these discourses. In total, we collected all material related to five Twitter discussions initiated by the government (1188 retweets and comments). Stage 2 of the data collection and analysis was intended to broaden our empirical settings by including the “citizens‐to‐citizens” discourse on Twitter (1014 tweets). We collected tweets over a period of 6 months (March to August 2020) and copied them into a spreadsheet. Each tweet was assigned an ID number and formed one unit of analysis.

*Coding*: Tweets were coded manually by the research team of two co‐authors, focusing on the contents of tweets. Coding reliability was ensured by team‐coding: A code was included in the dataset when a consensus in the interpretation was reached between the coders. To amplify the plausibility of interpretations, we undertook multiple rounds of analysis (Creswell, 2014), focusing on the meanings of the tweets (Lukka & Modell, 2010) rather than on the actual expressions and wordings used by the informants (Kozinets, 2015). Each tweet could have more than one code assigned to it.

In addition to the content, we coded the tone of each Twitter message to acknowledge the traces of emotional colorization (Maurer & Diehl, 2020). The choice of a specific tone indicates the feelings of an author toward an issue, thus revealing affirmation, indifference or opposition. The positive and negative tones were identified in the statements where sentiments were clearly shown by the authors of a tweet. Other tweets were coded as neutral. An example of a neutral message would be sharing some information about the CTA and accompanying it by a statement with no positive or negative emotional colorization. The following coding example shows a tweet with a negative tone and the assigned codes “decentralized,” “trust in government” and “Brexit”:

Did NHSX choose the centralised approach as it was: a) best b) told to Success depends on c.80% adoption [sic]. This won't be achieved without trust and we don't trust you or those Leave cronies you're in bed with. (#10010010; coded: ‘decentralised’; ‘trust in government’; ‘Brexit’; ‘negative tone’)

**FIGURE 1:**
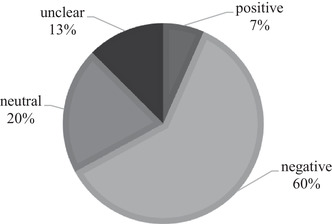
Semantic distribution

Figure 1 shows that our dataset contains 7% of statements with a positive, 20% of statements with a neutral and 60% of statements with a negative tone. Further, we identified 13% of statements with an unclear tone, for instance when messages shared publicly available secondary‐source information without any accompanying personal comment.

*Analysis*: We used discourse analysis to interpret and collate codes. This recognized method offers a combination of “textually orientated and social theory approaches” (Ferguson, 2007, p. 919) in which textual discourse “is conceived as having a dialectical relationship with social structure” (Dick & Cassell, 2002, p. 999). Such an approach emphasizes the meanings generated through public conversations (Atkinson, 2017; Johns et al., 2004).

Both members of the research team were involved in the analysis during all three stages. Stage 1 included data reduction and systematization through searching for emerging discursive themes and assigning meanings to these identified themes (Miles & Huberman, 1994; O'Dwyer, 2004). Stage 1 led to the identification of 15 initial themes characterizing the discourse. Stage 2 aimed to confirm the initial themes from Stage 1, as well as to identify additional themes; five additional themes were identified in this stage.

Both government‐to‐citizens and citizens‐to‐citizens discourses laid the groundwork for the study findings and were analyzed together in Stage 3. To enhance theorization, during this stage, the 20 emerging initial themes were collapsed into eight aggregated topics (Table 2). Four of the topics tended toward the technical side and the other four toward the social aspects of co‐production. The topics were subsequently interpreted and theorized. The analysis and interpretation were performed by mobilizing the analytical lens of UTAUT and the complementary social aspects of trust, accountability and social environment. The frequency of each aggregated topic's occurrence in the dataset was calculated in order to get a better understanding of the topics referred to in the debate (see Table 2). The themes beyond the eight aggregated topics (approximately 14% of the dataset) were separately recorded, with the majority of tweets referring to the situation of CTAs in other countries. During Stage 3, we checked for alternative interpretations to strengthen our analysis until both team members became convinced (O'Dwyer, 2004).

**TABLE 2:** Aggregated topic distribution and the frequency of occurrence

Technical aspects:Performance and effort expectancy		Social aspects:Trust, accountability and social environment	
Topic	Frequency	Topic	Frequency
(De)centralization	15.5%	Privacy and surveillance	16.1%
Performance and security	10.4%	(Dis)trust	12.6%
Individual choice and usability	9.6%	Accountability and transparency	9.5%
Effectiveness	6.3%	Freedom and misuse	5.9%

The insights from the analysis are presented in the next section. We structure our findings around the identified aggregated topics, starting with the ones which tend more toward the technical side, followed by those tending to the social aspects. We organize the presentation of our findings according to the frequency with which a particular topic occurred, beginning with the one with the highest frequency.

## 5 FINDINGS

Our analysis of the public discourse on Twitter revealed a high level of public interest in the proposed co‐production. The responses to the government tweets and citizens‐to‐citizens discussions showed the polarization within the observed discourse. This polarization was mainly driven by two factors. First, a delay in the availability of the app in comparison to other countries sparked the debate and boosted its longitudinal development. Second, the discourse related to the introduction of the CTA was interwoven with interrelated discussions of the UK government's general response to the emergency situation (given that the UK was the European country most severely affected by the pandemic at that time).

To answer our research question of how citizens’ perceptions affect the co‐production of digital public services in the context of an emergency, we examine the studied discourse by applying the rational‐technical lens of UTAUT, at the same time acknowledging simultaneously the roles of the social environment, trust of citizens in the government and the implications of trust for the accountability relationships between the parties. The subsections below outline our findings on the technical (5.1) and social aspects (5.2) of citizens’ perceptions of co‐production revealed in the discourse.

### 5.1 Technical aspects: Performance and effort expectancy

There are a number of (formal and rational) preconditions determining a new technology (such as an e‐government service) becoming accepted by potential users. First, it has to be regarded as providing a solution to a problem. Second, the effort to access and use the service needs to correspond to the benefits gained (Venkatesh et al., 2003). This subsection analyzes the topics related to the technological aspects of the UK CTA, including *(de)centralization, performance and security, individual choice and usability*, and *effectiveness*.

In a high number of the tweets analyzed, citizens expressed concerns about *centralization* as the government's initial approach, which involved storing all data gathered through the CTA on a central server and matching the contacts centrally in case an infection is reported. Many of these tweets had a negative tone revealing their authors’ skepticism and anxiety, as in this example:

Every IT security expert I know said that the UK Gov[ernmen]t's ‘centralised’ approach to the contact tracing app was disastrous. How many millions have been wasted before the Gov[ernmen]t finally made the inevitable U‐turn? Utterly incompetent (#20250005).

Acknowledging this criticism, the UK government eventually switched to the decentralized framework developed by Apple and Google, following the approach of most Western countries. However, although the shift to a decentralized framework was welcomed, citizens pointed out the additional cost brought about by the change in design of the CTA. In addition, the change in underlying technology led to a delay until the final version of the app was released (Kelion, 2020).

Our analysis showed that public concerns regarding *performance and security* acknowledged the importance of the app being free from technical malfunctions (such as errors, bugs and vulnerability to third‐party interventions). When the government published the source code for the trial version that was rolled out on the Isle of Wight and asked citizens to use and give their vires of the app, a number of comments were made that generally endorsed the approach taken. In addition, some feedback from citizens was posted regarding error messages. This signals citizens’ willingness to engage in the initial stage of co‐production by assisting the government in its attempts to deliver a new digital service.

However, the government was not able to mitigate the high level of concern expressed by citizens about the potential vulnerability of the app to data breaches and hacking attacks. A tweet on the use of Bluetooth technology for exchanging contacts reveals this anxiety:

[…] I have asked this before and had no response, but I'll ask again. Bluetooth is an insecure technology, what protections are there against bluesnarfing, bluejacking and bluebugging? (#10040022).

Besides these concerns, it was also pointed out that the Bluetooth interface had always to be activated in order for the app to function as intended, which led to complaints about the app consuming too much energy from the phone battery.

Effort expectancy, that is how difficult or simple a new digital service is to use for an individual, was manifested in the aggregated topic *individual choice and usability*. For instance, regarding the installation of the app, citizens confronted the fact that the CTA had only been developed for Android and iOS operating systems running on mobile phones, leaving behind citizens (especially, the elderly) who might use other systems:^2^

What about the Windows Phone code? Both my parents use Windows phones (#10050004).

Resolving this issue was pressing, alongside offering training to the population on how to use the app. Taking into account that the CTA was initially presented as a critical e‐government service in response to the pandemic, here our study draws attention to the classic challenge facing the UK government of a “digital divide” in e‐government (Sorrentino et al., 2018). A common strategy to overcome the “digital divide” is to offer government services both online and offline. However, such an approach was not available for the UK CTA, as there was no offline alternative to replace the exchange of anonymized data.

When it comes to the topic of *effectiveness*, citizens’ main attention was given to the question of whether the UK CTA could be an effective instrument to tackle the pandemic. In particular, citizens were concerned about whether they could trust other users, as it was essential to secure the trustworthiness of the data input. Having reliable data was seen as crucial for the app's success, but the potential for manipulation was highlighted at the same time:

Just make sure we don't have fools misusing the app sending false fears en‐masse they are COVID‐19 positive! (#10040075)

In addition, citizens argued that lockdowns could be avoided if adoption rates of CTAs were sufficiently high. However, they doubted whether the required rate of 60% (Findlay et al., 2020) could be achieved in principle.

Concluding here our overview of the public discussion related to the technological aspects of the proposed co‐production, our study reveals that a substantial number of messages focused on doubts, skepticism and anxiety. In particular, the initial plan to use a centralized approach was criticized—but also the subsequent change to a decentralized approach, as it was regarded as evidence of the poor overall planning of the CTA project. In addition, the inability of significant numbers of people to access the app was argued to manifest a “digital divide.” We continue by discussing the revealed topics in terms of the wider social aspects.

### 5.2 Social aspects: Trust, accountability and social environment

While the online debate revealed the importance of technical aspects of the CTA, social aspects received equal attention. From the analysis of themes, four aggregated topics emerged: *privacy and surveillance, (dis)trust, accountability and transparency* and *freedom and misuse*. The discussion about the CTA's contribution to public health policy and potential malfunctions was at times intense and heated. These four topics are analysed in this subsection.

The conditions of the social environment for the implementation of the CTA included fears about *privacy and surveillance*:

Don't blackmail us onto [sic] accepting your control grid surveillance state (#10010204).

A number of further tweets described scenarios of dystopia, outlining potentially mal/dysfunctional aspects of the CTA. For instance, there was fierce criticism that big data analytics could be used to monitor the population, for example for profiling and policing purposes. Also, stark disapproval was voiced of individuals’ inability to remove any of their personal data held in the centralized system:

Britons will not be able to ask NHS admins to delete their COVID‐19 contact‐tracking data from government servers […] Install the contact tracing app? Are you mad? (#20230002).

The government attempted subsequently to accommodate the articulated concerns by changing to a platform that does not store personal data on a central server and by publishing the source code of the app (but the front‐end only: see below). Transparency about the source code was communicated in a number of follow‐up tweets by the government.

The topic of public *(dis)trust* appeared frequently within our dataset. While a certain degree of skepticism toward the government can be regarded as an indication of vigilance, trust between the partners is a precondition for co‐production (Fledderus et al., 2014). The unwillingness of citizens to co‐produce was evidenced in tweets referring to explicitly to a lack of trust in the CTA digital government service. Often, such critical voices expressed general dissatisfaction with the handling of the COVID‐19 pandemic by the government (Cairney & Wellstead, 2020). For example, such opinions were articulated as follows:

No chance of putting app on phone; I DO NOT TRUST THE GOVERNMENT. THEY have lied about PPE, TESTING and DEATHS. Probably lying about the APP (#10050048).

The topic of *(dis)trust* was also observable when citizens expressed doubts about the effectiveness of the app and its security, and uncertainty about the effort expected from their side. Some statements showed disbelief that the government had the ability to protect citizens’ privacy and worries that citizens would be left without effective instruments to hold their government to account in the co‐production process. The expressions of lack of trust and of the anxiety experienced were amplified by the delay of the launch of the CTA, perceived as a further unfulfilled commitment of the government to its citizens.

Furthermore, our analysis revealed the positive and negative perceptions regarding *accountability and transparency* associated with the government's introduction of the UK CTA. The discourse showed that the government poorly specified and articulated the conditions of co‐production, for instance whether the app was supposed to play a central or just an additional role to manual contact tracing. Despite the demands for accountability that were conspicuous in the tweets during the trial phase, there were many complaints that the government failed to address these demands. Citizens also claimed that the app's development process was not open and transparent. Although the government eventually published the source code of the app as the front‐end of the CTA, the back‐end (server side) of the code was not published, which was criticized:

[W]hat about the rest of the system? The client is only half the story – where is the repo for the server code? (#10050032).

In addition, a lack of transparency was perceived in the procurement process for the CTA, and, as no tender was made, the possibility of corruption. For instance, despite the absence of any public announcements, citizens became aware of the government contracting Accenture, an external technology and consulting company, to deliver an unspecified range of services related to the UK CTA. Media outlets reported that the government was making use of an earlier framework contract and had not required a separate tender when hiring the firm for the app‐related services. This amplified the concerns observed in the discussion regarding the government's approach and the lack of transparency in its overall strategy for tackling the pandemic.

When it comes to the notions of *freedom and misuse*, our findings reveal a broad range of anxieties regarding the app being (potentially) vulnerable to misuse:

Contact tracing apps will serve as vehicles for abuse, disinformation and provide a false sense of security (#20300006).

Examples of potential misuse cited in other tweets included health insurance companies restricting cover where the app was not installed by insurance takers, (potential) employers requiring employees to install the app, or landlords discriminating against tenants who do not have the app installed.

A number of tweets both explicitly and tacitly demonstrated their authors’ belief that the app was vulnerable to both intentional and unintentional forms of misuse, which determined the hesitation to engage in co‐production. Therefore, even though the skepticism and concerns of citizens about the UK CTA have different roots and preconditions, they lead to one alarming outcome, expressed in the quote below, which brings into question the future of digital contact tracing in the UK:

Don't download the app if you value your future freedom (#10010159).

In sum, through our analysis, our case revealed that the (voluntary) co‐production of CTA is complex and multifaceted. Technical issues, public health considerations, individual freedom, the requirement of widespread adoption of the app for effectiveness and accountability concerns all play a role. In particular, the analysis shows that the government was not clear from the start about the role of the app, and correspondingly, about the roles that citizens were supposed to play in co‐production. Also, despite conspicuous demands for accountability, citizens felt left without the means to hold the government accountable. In the following section, we discuss the wider implications of our findings.

## 6 DISCUSSION AND CONCLUSION

During the global COVID‐19 pandemic governments rushed to deploy a wide range of measures, including the e‐government solution of CTAs. Our study aimed to explore how public scrutiny and citizens’ concerns affect the (voluntary) co‐production of digital technologies in the public sector in the midst of an emergency situation, looking at the case of the UK's CTA. Our study contributes to research on social aspects of co‐production of digital services, including accountability, trust and users’ beliefs (Fledderus et al., 2014; Loeffler & Bovaird, 2019). It examines the contextually rich case of a service that can only be effective when participation in co‐production is substantial at a societal level, yet where non‐participation is not sanctioned. In this section, we discuss our findings and outline the broader implications of our analysis.

*Co‐production*: First, our study adds to the growing body of research on co‐production to combating COVID‐19 that takes multiple angles on the topic, such as community‐centered approaches (Cepiku et al., 2020), citizen‐state collaboration (Zhao & Wu, 2020), lessons from co‐production in emerging economies (Turk et al., 2021) and asking about the persistence of co‐produced solutions in a postpandemic time (Steen & Brandsen, 2020). Despite recent research claiming that co‐production has been “blooming under Covid‐19” and that social distancing measures, for example, “could be regarded as a gigantic co‐production project” (Steen & Brandsen, 2020, p. 852), the CTA users’ concerns that we report on reveal the need for a deeper understanding of the socioenvironmental preconditions for and the setting of co‐production. We observed that the barrier to participation is high when sensitive data is involved. In such a sensitive and highly personal context, concerns about the potential misuse of data led to substantial public skepticism of and distrust in the proposed digital service. Although encouraged to participate in the government's innovation with the proclaimed aim to “save lives” as active (and equal) partners in a process of co‐production, citizens found they had little influence on how to protect and manage their own data once they provided it as part of their contribution to the co‐production. Our study evidences citizens’ perceptions of the fundamental vulnerability of digital services such as a CTA, which might “be used […] against the people it's designed to protect” (Privacy International, 2020). With this, we contribute to extending the commonly applied UTAUT (Venkatesh et al., 2003) by pointing out the boundary conditions for citizens’ engagement in co‐production (Alford, 2002; Farronato et al., 2020) and contextualizing the complexity of socioenvironmental settings of the co‐production in times of a global pandemic.

*Accountability*: Second, our study acknowledges the importance of understanding the role of public discourses on accountability and trust in the co‐production of digital public services (Jayasinghe et al., 2020), looking at the challenging context of addressing the pandemic, while preserving democratic freedoms. We consider online public discourses on accountability, trust and other matters important to the public as valuable repositories (Jeacle & Carter, 2011; Neu et al., 2019) that reveal insights into how individuals use written communication “to produce explanations of themselves […] and the world in general […], as alternative discourses […] enable individuals and groups to resist the regulatory norms in any specific social domain” (Cassell & Symon, 2004, pp. 203–204). Close monitoring of government action and calling for an accountability dialogue can be seen as a sign of a vigilant society (Van De Walle & Six, 2013). Our study sheds light, in particular, on the accountability discourse revealing the dilemma of public safety versus individual freedoms in the context of the new instruments of individual tracing. We demonstrate how the citizens’ perceptions of the UK government's attempts at accountability as insufficient and unsatisfactory caused the failure of co‐production despite the initial engagement of citizens by contributing their views on the technological advancement of the service. The study thus adds empirical evidence from the context of an emergency situation to support the proposition that alongside carefully planned digital innovations, strategic accountability conduct is a crucial determinant for stakeholder (co‐producer) engagement (Cooper & Lapsley, 2019) and subsequent outcomes of the proposed co‐production. Having said that, the circumstances of an emergency situation and the CTA as a digital service delivered “externally” to the public might be a particular context in which digitalization evolves. We thus encourage further research to examine and re‐visit the central relationships identified in this study in different settings, for example focusing on the implementation of digital technologies for “internal use” in public organizations.

*Technology acceptance*: Third, our study emphasizes the importance of social aspects and boundary conditions for the acceptance of technology in the public sector. When looking at the Twitter debate in the UK, we found that technical issues were indeed present. However, these were intermingled with socioenvironmental issues of particular importance, such as the general government response to the pandemic (see also Mansoor, 2021). We acknowledge that this observation is grounded in a country‐specific empirical setting from a country with a low general level of citizen satisfaction with the government's response to the pandemic (Cairney & Wellstead, 2020; Devlin & Connaughton, 2020). Alongside this, we suggest that the success or failure of a CTA needs to be seen in the light of the implementing government's overall emergency response, e‐government strategy and relevant policies. Building on our findings, we contend that the citizen's willingness to co‐produce should not be considered in isolation (i.e., for a single service), but in the light of other government actions and services, such as political developments and the wider “service system” (Osborne et al., 2016).

Even though the pandemic provides a fertile empirical setting, it also limits this study in its ability to capture the longitudinal development of the individual rationales underlying citizens’ opinions as expressed on Twitter. Research needs to advance understanding of the longitudinal dynamics of digital contact tracing in various (inter)national settings and the long‐lasting effects and accountability implications of CTAs, which could be scrutinized based on further developments over time. In addition, we encourage future researchers to study different settings of co‐production within their socioeconomic complexity and their potential to enhance the level of interaction with the users of digital public services (Osborne et al., 2021). For exploring this research terrain further, the richness of the empirical context at the intersection between digital services, co‐production and emergencies could be used to learn more about governmental accountability for the roll‐out of new applications in the context of an unprecedented technological intervention in the lives of individuals.

## CONFLICT OF INTEREST

The authors declare no conflict of interest.

## Data Availability

The data that support the findings of this study are available from the corresponding author upon reasonable request.

## Notes

1 Hosted the UK CTA's trial.

2 Other systems include conventional phones unable to install apps and smartphones running on a Windows operating system or a dated operating system.
